# Delayed Thrombin Generation Is Associated with Minor Bleedings in Venous Thromboembolism Patients on Rivaroxaban: Usefulness of Calibrated Automated Thrombography

**DOI:** 10.3390/jcm9072018

**Published:** 2020-06-27

**Authors:** Jaroslaw Zalewski, Konrad Stepien, Karol Nowak, Sandi Caus, Saulius Butenas, Anetta Undas

**Affiliations:** 1 Department of Coronary Artery Disease and Heart Failure, Jagiellonian University Medical College, John Paul II Hospital, 80 Pradnicka Street, 31-202 Krakow, Poland; konste@interia.eu (K.S.); karol.nowak@student.uj.edu.pl (K.N.); 2 Department of Biochemistry, University of Vermont, 89 Beaumont Avenue, Burlington, VT 05405-0068, USA; sandi.caus@uvm.edu (S.C.); sbutenas@uvm.edu (S.B.); 3 Department of Experimental Cardiac Surgery, Anesthesiology and Cardiology, Institute of Cardiology, Jagiellonian University Medical College, 80 Pradnicka Street, 31-202 Krakow, Poland; mmundas@cyf-kr.edu.pl

**Keywords:** venous thromboembolism, minor bleeding, calibrated automated thrombography, rivaroxaban, vitamin K antagonist

## Abstract

Bleeding is the most feared and difficult to predict adverse event of anticoagulation. We sought to investigate whether calibrated automated thrombography (CAT) parameters are associated with minor bleeding (MB) in anticoagulated patients following venous thromboembolism (VTE). Enrolled were 132 patients on rivaroxaban, 145 on vitamin K antagonists (VKA) and 31 controls who stopped anticoagulation. Prior to the next dose of the anticoagulant, we measured CAT parameters, along with rivaroxaban concentration and INR. During a median follow-up of 10 months, we recorded minor and major bleedings. On rivaroxaban, 27 (20.5%) patients with MB had longer time to start thrombin generation, lower peak thrombin generation and lower endogenous thrombin potential compared with subjects without MB (all *p* < 0.001). All CAT parameters, except for peak thrombin generation (*p* = 0.049), were similar in VKA patients with (*n* = 25, 17.2%) vs. without MBs. By logistic regression, time to start thrombin generation (*p* = 0.007) and unprovoked VTE (*p* = 0.041) independently predicted MBs on rivaroxaban. Major bleedings were more frequent in patients with MBs (17.3% vs. 1.8%, *p* < 0.001). Abnormal CAT parameters characterize VTE patients prone to MBs on rivaroxaban, but not on VKA. Time to start thrombin generation measured about 24 h since the last rivaroxaban dose might help predict MBs.

## 1. Introduction

Minor bleeding (MB) is usually defined, based on the International Society on Thrombosis and Hemostasis (ISTH) guidance, as a clinically overt event not meeting the criteria of major or clinically relevant non-major (CRNM) bleeding [1,2]. MB represents a common, unappreciated and poorly understood adverse event in patients receiving anticoagulation. The RIETE [3], GARFIELD-VTE [4] and XALIA [5] registries performed in VTE patients treated with rivaroxaban, as well both EINSTEIN trials with rivaroxaban in patients with symptomatic deep-vein thrombosis (DVT) [6] and those with pulmonary embolism (PE) [7], have not specifically reported MBs. A high prevalence of MBs have been reported in observational studies. Själander et al. found increased incidence of easy bruising from 17.8% to 75.6%, menorrhagia from 44.2% to 70.8%, gingival bleedings from 22.2% to 48.3%, bleedings after tooth extraction from 3.0% to 45.2% and epistaxis from 11.1% to 23.6% after intiation of anticoagulation with vitamin K antagonist (VKA) among women [8]. In turn, in VTE patients treated mostly with non-vitamin K antagonist oral anticoagulants (NOAC) MBs were frequently found including heavy menstrual bleeding in 23.2%, easy bruising in 15.4% and gingival bleeding in 8.8% of subjects [9,10]. The Dresden NOAC registry [11] showed that ISTH-defined MBs occur with the frequency of 37.8 per 100 person-years in real-life VTE subjects treated with rivaroxaban and they accounted for 58.9% of all observed bleedings. In turn, MBs in VTE patients on apixaban or rivaroxaban defined as outpatient claims not requiring hospital admission were reported in 20 or 34 per 100 person-years, respectively [12].

It has been suggested that MB might predict subsequent major adverse events in patients on chronic anticoagulation. Veeger et al. [13] found that in patients on VKA, MBs were associated with a 3-fold increased risk of subsequent major bleeding as compared with the remainder, and this relationship was independent of quality of anticoagulation. Moreover, in a large cohort of patients on VKA, Van Rein et al. [14] demonstrated that increased risk of major bleeds in patients following MBs is due to fixed and currently unknown underlying risk factors. In patients with AF, occurrence of only MBs on apixaban or VKA was associated with higher risk of subsequent major bleeding (hazard ratio (HR) 1.53, 95% confidence interval (CI) 1.37–2.26), ischemic stroke (HR 1.69, 95% CI 1.09–2.61) and all-cause mortality (HR 1.25, 95% CI 1.02–1.55) [15]. Evidence for similar minor–major bleeding relationships in VTE patients treated with NOAC is not available.

Calibrated automated thrombography (CAT) parameters reflect the overall function of the blood clotting system. Increased endogenous thrombin potential (ETP) and peak thrombin concentration, together with shorter time to thrombin peak, reflect an enhanced and faster activation of blood coagulation and is typical of a prothrombotic state in vivo [16]. In turn, a decreased thrombin generation predicts a bleeding tendency in patients with von Willebrand disease [17] and with hemophilia A [18]. This assay was also used to evaluate the effect of prothrombin complex concentrates in reversing the anticoagulant effect of rivaroxaban [19,20]. CAT profiles used for many years to assess the overall function of the blood clotting system [21,22], are significantly modified by anticoagulants, including NOAC [23,24]. Both rivaroxaban and warfarin effectively inhibit thrombin generation in patients with VTE as evidenced by reduced ETP, peak thrombin as well as prolonged lag time and time to thrombin peak, as compared to normal controls [25]. However, their impact on individual CAT parameters differs. Rivaroxaban, a direct inhibitor of factor Xa, mainly influences the initiation and propagation phases of thrombogram, which leads to the protraction of thrombin generation curves [23]. As a result, there is a lower than expected decrease in ETP. In turn, VKA reducing the activity of vitamin-K-dependent coagulation factors have an uniform and generalized effect on all CAT parameters [23,26].

In this study, we tested the hypothesis that CAT parameters may help identify anticoagulated VTE patients prone to developing bleeding tendencies during long-term treatment with rivaroxaban, the most commonly used NOAC in VTE patients, as well as with VKA.

## 2. Materials and Methods

We screened 353 consecutive adult patients with documented symptomatic VTE aged from 22 to 71 years, referred to outpatient clinics between January 2012 and January 2016. The diagnosis of DVT was established on the basis of positive findings on color duplex ultrasound, whereas the diagnosis of PE was performed by positive result of high resolution computed tomography. Patients with a history of portal vein thrombosis or cerebral sinus vein thrombosis confirmed by imaging were also eligible. Following documented VTE episodes, all patients were treated with unfractionated or low molecular weight heparins followed by VKA or rivaroxaban, for at least six months in patients with VTE provoked by transient risk factor, or longer in patients with unprovoked VTE. The decision as to whether VKA or rivaroxaban would be continued was left at the discretion of the treating physician based on the patient preferences. A VTE episode was defined as provoked if the patient had a history of surgery requiring general anesthesia, major trauma, pregnancy or delivery, hospitalization within the prior three months, hormone replacement therapy or oral contraception. A proximal DVT was defined as the presence of thrombus in the popliteal, femoral or iliac veins.

We excluded patients diagnosed with cancer, chronic kidney disease (CKD) stage 4 and 5, acute coronary syndrome or ischemic stroke within the previous 6 months, any acute infection, all the states known to affect thrombin generation [21] or with the time since last dose of rivaroxaban of less than 20 h. All patients on anticoagulation were asked to show up prior to administration of the next dose of the anticoagulant. Of 353 VTE patients, 45 patients were excluded from the analysis because of unavailable CAT data or follow-up data (*n* = 21) or a time interval of <20 h since the last rivaroxaban dose (*n* = 24).

Obesity was defined as a body mass index (BMI) over 30 kg/m^2^. Diabetes mellitus was stated as a history of diabetes, use of antidiabetic agents, or fasting plasma glucose ≥126 mg/dL (7 mmol/L) on two separate occasions. Hypertension was defined as office systolic blood pressure values ≥140 mmHg and/or diastolic blood pressure values ≥90 mmHg or current antihypertensive treatment. Chronic kidney disease stage 3 was diagnosed when creatinine clearance, calculated using the Cockcroft–Gault formula, was lower than 60 mL/min. Heart failure referred to a symptomatic condition with relevant structural heart disease with diastolic dysfunction or reduced left ventricular ejection fraction below 40%.

The study protocol complied with the Declaration of Helsinki and was approved by the Bioethical Commission of Local Medical Chamber (approval number 135/KBIL/OIL/2013). All included patients gave informed consent before they participated in the study.

### 2.1. Plasma Preparation

Fasting blood samples were drawn between 8 and 10 a.m. from an antecubital vein directly into tubes containing citrated anticoagulant after at least 3 months of anticoagulation. The blood samples were centrifuged at 2.500 g at a temperature of 18 °C to 22 °C for 20 min and processed immediately or stored in aliquots at −80 °C until analysis.

### 2.2. Laboratory Measurements

The laboratory investigations, including international normalized ratio (INR) and activated partial thromboplastin time (APTT), were assayed by routine hospital techniques. The quality of anticoagulation with VKA was assessed by time in the therapeutic range according to the method of Rosendaal (TTR). Fibrinogen was determined using the Clauss assay. High-sensitivity C-reactive protein (CRP) was measured by immunoturbidimetry (Roche Diagnostics GmbH, Mannheim, Germany). Plasma D-dimer was measured with the Innovance D-dimer assay (Siemens, Marburg, Germany).

### 2.3. Thrombophilia Testing

Thrombophilia screening was performed in all study participants. Factor V Leiden and prothrombin G20210A polymorphisms were determined using TaqMan Genotyping Assays (Assay ID: C_11975250_10 and C_8726802_20, respectively; ThermoFisher Scientific, Waltham, MA, USA) on QuantStudio Dx Real-Time PCR Instrument (ThermoFisher Scientific) as described previously [27]. Plasma Protein C activity was quantified using a chromogenic assay (HemosIL Protein C Instrumentation Laboratory, Milan, Italy or Berichrom Protein C, Siemens Healthcare Diagnostics). Free protein S levels were measured with an immunoturbidimetric assay (INNOVANCE^®^ Free PS Ag, Siemens Healthcare Diagnostic) [28]. Antithrombin activity was measured using an assay based on FXa inhibition (INNOVANCE™ ATIII, Siemens Healthcare Diagnostics, Marburg, Germany) [29]. Antiphospholipid syndrome was diagnosed according to the current recommendations [30]. All coagulation tests were performed twice and yielded positive results.

### 2.4. Rivaroxaban Concentration

Rivaroxaban concentration was measured by the anti-Xa chromogenic assay, Biophen DiXaI (Hyphen Biomed, Neuilly-sur-Oise, France) according to the manufacturer’s instructions. Briefly, 200 µL of diluted plasma (1:8 with Tris-NaCl EDTA buffer at pH 7.85) were incubated with 50 μL of human FXa (Hyphen BioMed, Neuilly-sur-Oise, France) for 120 s at 37 °C, then 50 μL of a specific FXa substrate were added to start the reaction and the color development was measured at 405 nm [31,32]. The calibration was performed with standards for low plasma concentrations of rivaroxaban (Biophen Rivaroxaban Calibrator, Neuilly-sur-Oise, France) [33].

### 2.5. Thrombin Generation Assay

The thrombin generation assay was performed as previously described [21,22]. The assay was performed in untreated, polystyrene 96-well plates (Costar, Lowell, MA, USA). Citrated plasma samples were thawed at 37 °C for 3 min and 5 mg/mL corn trypsin inhibitor was immediately added to achieve a 0.1 mg/mL final concentration. Eighty μL of each plasma sample, in duplicate, was added to a 96-well plate followed by addition of relipidated tissue factor at a final concentration of 5 pM. The fluorogenic substrate used was benzyloxycarbonyl-Gly-Gly-Arg-7-amido-4methyl-coumarin·HCl (Z-GGR-AMC) (Bachem, Torrance, CA, USA). Twenty μL of a 2.5 mM Z-GGR-AMC/90 mM CaCl_2_ solution in HEPES-buffered saline was added to plasma samples to achieve final concentrations of 417 μM and 15 mM, respectively. A 3 min incubation period at 37 °C followed to allow for recalcification of the plasma. Twenty μL of a 120 μM phospholipid vesicle solution (25% dioleoyl-*sn*-glycero-3-phospho-l serine and 75% 1,2-dioleoyl-*sn*-glycero-3-phosphocholine) (Avanti Polar Lipids, Inc, Alabaster, Al, USA) in HEPES-buffered saline was then added to plasma samples to achieve a final concentration of 20 μM, thus initiating thrombin generation. Fluorescence readings began immediately and hydrolysis of the AMC (7-amino-4-methylcoumarin) substrate (at 370 nm excitation and 460 nm emission wavelengths) was followed over a 60 min period. Changes in fluorescence were converted to thrombin concentration using a calibration curve built by sequential dilutions of human thrombin (Haematologic Technologies, Inc., Essex Junction, VT, USA). The plate reader used was the BioTek Synergy 4 and analysis was performed using the Gen5 plate reader software (BioTek, Winooski, VT, USA). The CAT parameters including Lag time (s, time to start thrombin generation), TTP (s, time to the highest rate of thrombin generation), max IIa (nM, peak thrombin generation), max rate (nM/s, the highest rate of thrombin generation) and ETP (nM × s, endogenous thrombin potential, calculated as the area under the thrombin generation curve) were calculated.

### 2.6. Follow-up

All patients were assessed in the outpatient clinic every 6 months or by phone. They were instructed to report symptoms that suggested recurrent VTE or bleeding requiring appropriate confirmatory diagnostic and/or laboratory tests. We recorded MB defined as bleeding, not requiring outpatient visits, medical interventions or hospitalizations including easy bruising, skin ecchymosis, conjunctiva bleeds, gingival bleeds, epistaxis, haematuria, or vaginal bleeds. Easy bruising was defined as the self-reported, frequent (on 2 or more separate occasions) occurrence of bruises of at least 2.5 cm in diameter as well as the presence of one or more bruises on the day of any clinic visit during follow-up confirmed by an investigator. Major bleedings were defined according to the ISTH definition [1,2]. We also recorded recurrent symptomatic VTE. The diagnosis of recurrent symptomatic DVT was established based on positive findings of color duplex ultrasonography. In cases of suspected DVT recurrence in the same leg, non-compressibility of a previously compressible venous segment or an increase of at least 4 mm in the residual diameters was applied to confirm the diagnosis. PE was each time confirmed by computed tomography angiography.

### 2.7. Statistical Analysis

The study was powered to have a 90% chance of detecting a 15% difference in lag time between patients with and without MB using a *p* value of 0.05. Based on the CAT values in the previous study [31], in order to demonstrate such a difference or greater, 23 patients were required in each group. For a *p* value of 0.001, 45 patients per group were required. The power analysis was done based on a two-tailed *t*-test.

Statistical analyses were performed with IBM SPSS Statistics version 25 software. Continuous variables are expressed as mean ± standard deviation or median and interquartile range (IQR), whereas categorical variables are expressed as numbers and percentages. Continuous variables were first checked for normal distribution by the Shapiro–Wilk test and compared by Student t test when normally distributed or by the Mann–Whitney U test for non-normally distributed variables. Categorical variables were compared by the Fisher’s exact test. The Pearson or Spearman rank correlation coefficients were calculated to test the association between two variables with a normal or non-normal distribution, respectively. Differences in CAT parameters between groups were adjusted for rivaroxaban concentration or INR in patients on VKA with regression analysis. Receiver operating characteristic (ROC) curves were used to determine the optimal cut-off value of CAT parameters and their sensitivity and specificity in prediction of minor bleedings. All independent variables with their potential for confounding both the exposure and the outcome and a lack of significant correlation with other variables were included in the multivariate logistic regression model to determine independent predictors of minor bleeding. A two-tailed *p*-value of less than 0.05 was considered statistically significant.

## 3. Results

### 3.1. General Characteristics

In this study, 308 (87.3%) VTE patients were enrolled. Of them, 132 (42.8%) were on rivaroxaban, and 145 (47.1%) on VKA (77 on warfarin and 68 on acenocoumarol), while 31 (10.1%) patients following provoked VTE who stopped anticoagulation at least three months before inclusion served as controls. Baseline characteristics of the studied groups are shown in Table 1. One hundred and nineteen (38.6%) subjects had a history of symptomatic DVT alone, 74 patients had PE (24.0%) alone, 95 (30.9%) patients DVT combined with PE, and 20 (6.5%) patients had thrombosis of atypical location including portal vein thrombosis and cerebral venous sinus thrombosis. In 172 (55.8%) patients, VTE was unprovoked, whereas among provoked VTE patients, 37 (12.0%) subjects had VTE related to surgery or trauma, 23 (7.5%) to pregnancy, and 61 (19.8%) to hormone use.

Thrombophilia screening yielded positive results in similar proportions of patients on rivaroxaban and VKA, including factor V Leiden (22.7 vs. 15.9%, *p* = 0.17), prothrombin G20210A (4.5 vs. 7.6%, *p* = 0.33) and deficiencies in natural anticoagulants (9.1 vs. 15.9%, *p* = 0.11), respectively. There were no differences in the distribution of thrombophilias between patients with and without MB on rivaroxaban, while in VKA patients prothrombin G20210A mutation was more frequent in subjects with MB as compared to those free of MB (Table 1). The median time of anticoagulation with rivaroxaban was 8 (interquartile range, 5–15) months, while that for VKA was 9 (6–16) months (*p* = 0.47).

### 3.2. Bleeding and Thromboembolic Events During Follow-up

During a median follow-up of 10 (interquartile range, 7–19) months, MBs were observed in 27 (20.5%) patients on rivaroxaban, in 25 (17.2%) on VKA and in 2 (6.5%) controls (Table S1). In patients on rivaroxaban, major bleedings occurred in seven (5.3%) subjects, including five gastrointestinal bleeds and one menorrhagia. In five patients (71.4%) major bleeding was observed in individuals who also reported MB. In VKA patients, major bleedings occurred in six (4.1%) subjects, including two gastrointestinal bleeds and two vaginal bleeds (Table S1). In four VKA patients (66.7%), major bleed was preceded by MB. In the whole anticoagulated group, major bleeding was observed more often in patients reporting MBs (9 (17.3%) vs. 4 (1.8%), *p* < 0.0001) with risk ratio of 9.7 (95% CI 3.1–30.4).

Recurrent thromboembolic events were reported in five (3.8%) patients on rivaroxaban and in three (2.1%) on VKA, without any relationship with MBs. Irrespective of the anticoagulant treatment, there were no differences in CAT parameters between patients with and without recurrent VTE.

### 3.3. Minor Bleeding on Rivaroxaban

Subjects treated with rivaroxaban, who experienced MBs or not, were similar in terms of demographics, clinical and laboratory variables, including the duration of anticoagulation, aspirin use, and fibrinogen levels (Table 1). Patients with MBs more often experienced unprovoked VTE as compared with subjects without MB (88.9 vs. 53.3%, *p* < 0.001). A plasma rivaroxaban concentration was measured in 116 (87.9%) patients and CAT parameters in this group were not different from those in whom this concentration was unavailable, except for ETP, which was larger (150472 (129249–169270) vs. 132293 (77811-156610) nM × s, respectively, *p* = 0.003). A median time since last dose of rivaroxaban in patients with vs. without MB was similar (23 (20–24) vs. MB 24 (24–26) hours, respectively; *p* = 0.27). There was also no difference in rivaroxaban concentrations between patients with MB (35 (6–66) μg/L) and without this complication (25 (13–44) μg/L, *p* = 0.73).

**Figure 1:**
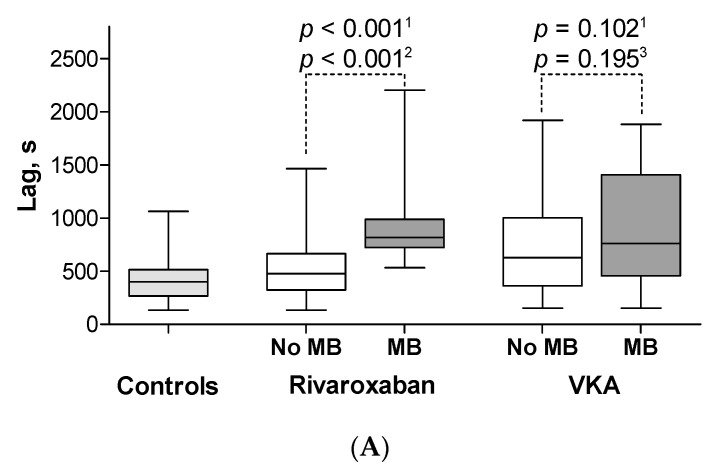

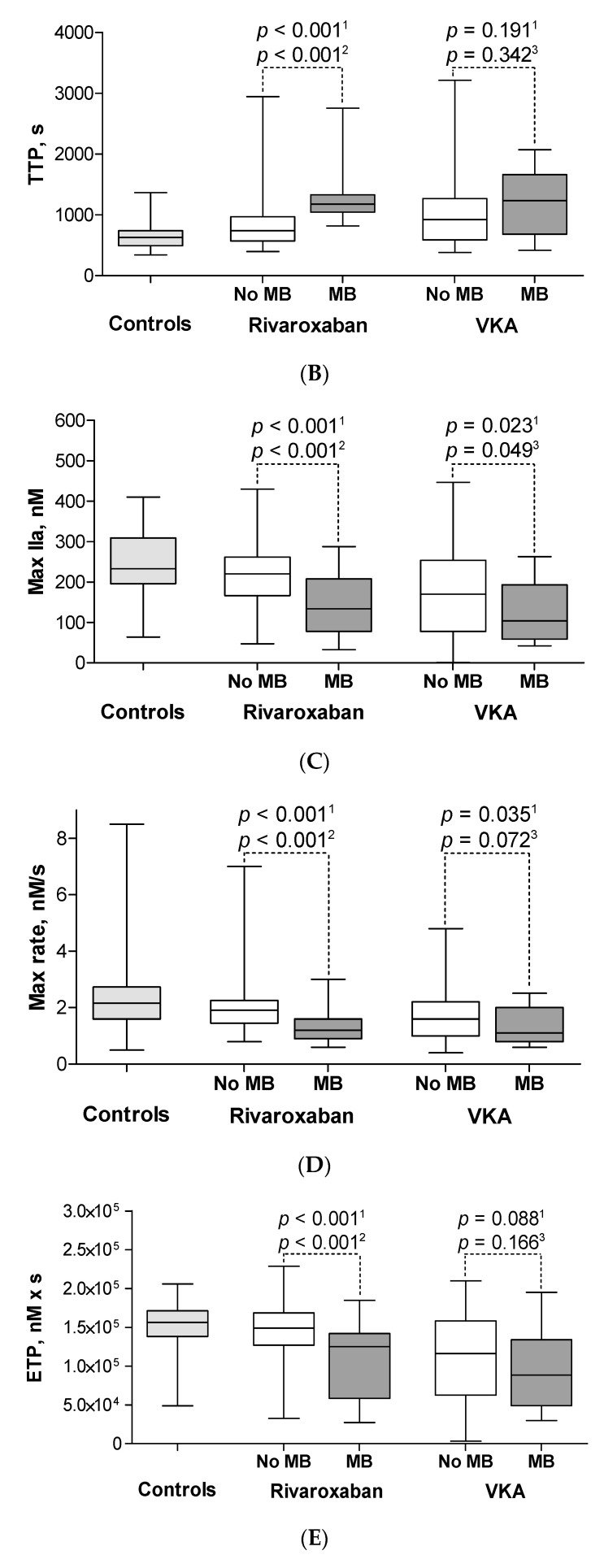
The calibrated automated thrombography parameters in the studied groups. Patients on rivaroxaban with MB had longer lag time (**A**), longer TTP (**B**), lower max IIa (**C**), lower max rate (**D**) and lower endogenous thrombin potential (**E**) as compared with subjects without MB. Patients on VKA with MBs had similar lag time (**A**), TTP (**B**), max rate (**D**), ETP (**E**) and lower max IIa (**C**) as compared with subjects without MB. Abbreviations: box plot shows median and interquartile range (IQR). Whiskers are drawn at Q3 + 1.5 × IQR, Q1−1.5 × IQR, MB: minor bleeding, VKA: vitamin K antagonist, lag time: time to start thrombin generation, TTP: time to peak thrombin generation, max IIa: peak thrombin generation, max rate: the highest rate of thrombin generation, ETP: endogenous thrombin potential, ^1^ Before adjustment differences compared by Student *t* test or by the Mann–Whitney *U* test and after adjustment with regression analysis for ^2^ rivaroxaban concentration or for ^3^ INR.

**Figure 2:**
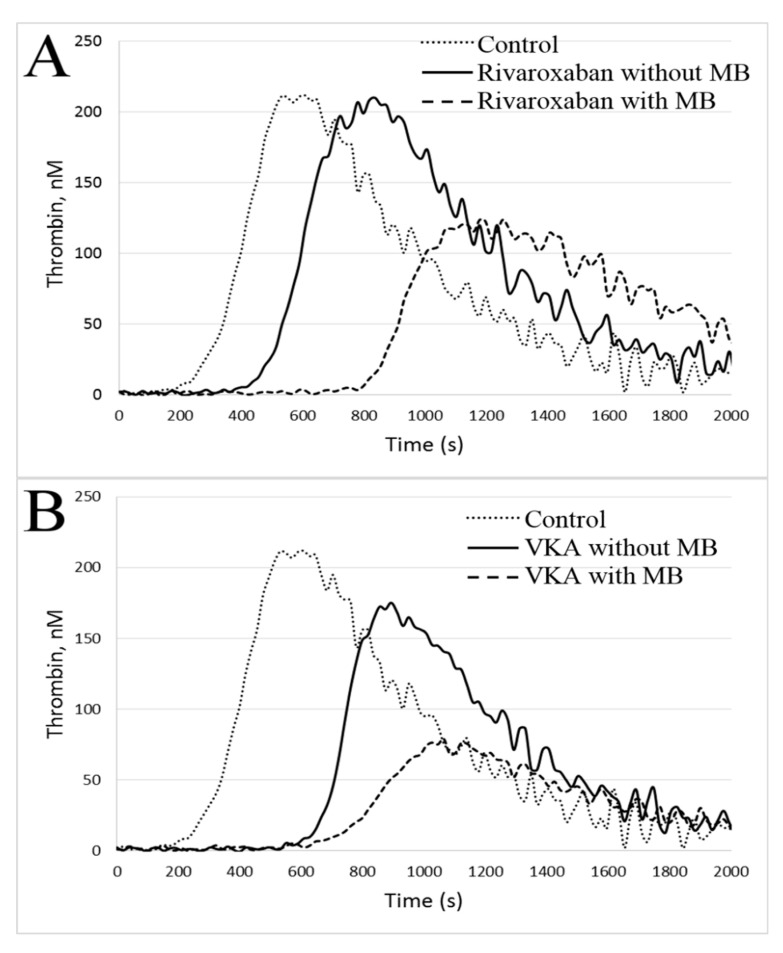
Representative thrombin generation curves in the calibrated automated thrombogram. (**A**). Rivaroxaban vs. control, (**B**). VKA vs. control. Abbreviations: MB: minor bleeding, VKA: vitamin K antagonist.

In the studied patients, there were no correlations between rivaroxaban concentrations and CAT parameters with the exception of TTP (*r* = 0.27, *p* = 0.02). After adjustment for rivaroxaban concentration, patients on rivaroxaban with MB had longer lag time (*p* < 0.001), longer TTP (*p* < 0.001), lower max IIa (*p* < 0.001), lower max rate (*p* < 0.001) and lower ETP (*p* < 0.001) as compared with subjects without MB (Figure 1, Table S2). Representative thrombin generation curves are shown in Figure 2.

In 40 patients on rivaroxaban, CAT parameters were measured twice after 3–6 months. As expected, there was a correlation between repeated CAT parameters, including Lag time (*r* = 0.48), max IIa (*r* = 0.51), ETP (*r* = 0.37), TTP (*r* = 0.33) and max rate (*r* = 0.32) (for all *p* < 0.05).

Baseline characteristics of patients on rivaroxaban without MBs and controls who stopped anticoagulation were comparable. There were no differences in CAT parameters between those two groups except for TTP (*p* = 0.025) (Figure 1, Table S2).

Patients on rivaroxaban with unprovoked VTE were significantly older, more frequently male, had higher BMI, hemoglobin and creatinine as well as a greater prevalence of deficiencies in natural anticoagulants (20.8 vs. 1.3%, *p* < 0.001) as compared with the provoked VTE group (Table S3). Patients with unprovoked VTE treated with rivaroxaban had lower max IIa and ETP as compared with those with provoked episodes (Table S3).

### 3.4. Minor Bleeding on VKA

MBs were reported by 25 patients (17.2%) on VKA, including 9 (11.7%) on warfarin and 16 (23.5%) on acenocoumarol (*p* = 0.08). MBs on VKA was not associated with patient characteristics, including aspirin use, except for the overrepresentation of individuals diagnosed with type 2 diabetes (16.0 vs. 4.2%, *p* = 0.048; Table 1). Laboratory investigations showed higher INR in patients with MB as compared with subjects without MB (2.19 (1.46–2.45) vs. 1.47 (1.02–2.20), respectively, *p* = 0.01), however the TTR values were almost identical in both groups (60% (43–80%) vs. 60% (46–80%), respectively, *p* = 0.81).

After adjustment for INR, patients treated with VKA with and without MBs had comparable lag time, TTP time, max rate and ETP. Only lower max IIa (*p* = 0.049) was found in patients with MBs as compared with the remaining subjects on VKA (Figure 1, Table S2). In groups with INR < 2 or INR ≥ 2, CAT parameters were not different between MB and non-MB subjects. There were significant differences between all CAT parameters of patients on VKA without MBs as compared with controls who stopped anticoagulation (Figure 1, Table S2).

### 3.5. CAT Parameters in Patients with Major Bleeding

In 13 (4.7%) patients with major bleeding on rivaroxaban or VKA, lag time was 703 (413–1302) s, TTP was 950 (646–1501) s, max IIa was 130 (60–225) nM, max rate was 1.1 (0.9–2.2) nM/s and ETP was 125101 (44445–143410) nM × s, without any differences as compared to the subjects with MB on rivaroxaban or VKA. There was a lower max IIa (*p* = 0.039) in patients who suffered from major bleeding during follow-up as compared with those free of any bleeding event. No other differences in CAT parameters were noted.

### 3.6. ROC Curves

**Figure 3:**
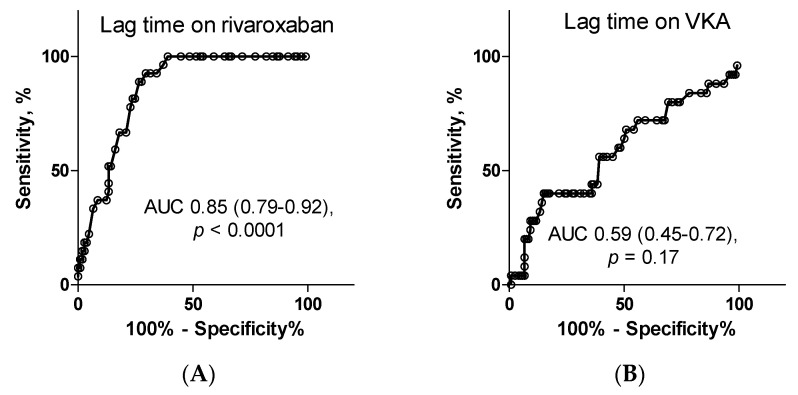

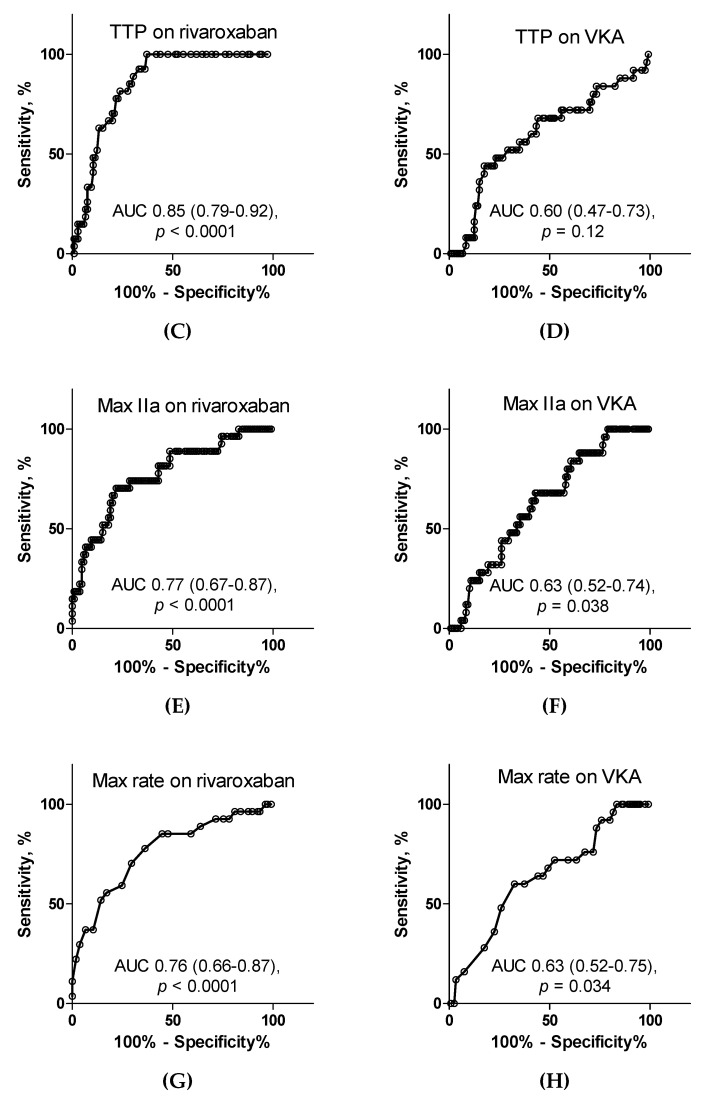

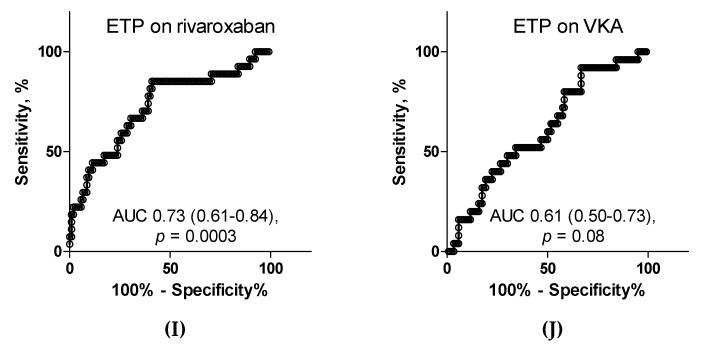
The receiver operating characteristics curves for calibrated automated thrombography parameters in prediction of minor bleedings. In patients on rivaroxaban, the highest predictive value for minor bleedings had lag time (**A**), TTP (**C**), max IIa (**E**) and max rate (**G**) and moderate ETP (**I**). In patients on VKA, moderate predictive values reached max IIa (**F**) and max rate (**H**) whereas lag time (**B**), TTP (**D**) and ETP (**J**) had no predictive value. Abbreviations: AUC: area under the curve, VKA: vitamin K antagonist, lag time: time to start thrombin generation, TTP: time to peak thrombin generation, max IIa: peak thrombin generation, max rate: the highest rate of thrombin generation, ETP: endogenous thrombin potential.

In patients on rivaroxaban, both lag time and TTP predicted MBs with the area under the ROC curve of 0.85 (*p* < 0.0001 for both; Figure 3). The cut-off value in prediction of MBs for lag time was 693.5 s with sensitivity of 81.5% and specificity of 76.2%, while the cut-off value for TTP was 1017 s with sensitivity of 77.8% and specificity of 77.1% and for max IIa was 173.5 nM (sensitivity, 74.1% and specificity, 71.4%) and for max rate was 1.55 nM/s (sensitivity, 70.4% and specificity, 70.5%). Finally, the corresponding values for ETP were 132772 nM × s, 66.7% and 69.5%. 

In patients on VKA, the highest, but moderate predictive values reached a max IIa and max rate with the area under the ROC curve of 0.63 (*p* < 0.05 for both) and both sensitivity and specificity did not exceed 60% (Figure 3).

### 3.7. Predictors of Minor Bleedings

In patients on rivaroxaban, age, gender, BMI, creatinine level, INR, rivaroxaban concentration, unprovoked VTE and CAT parameters were identified as potentially associated with MBs. By multivariate analysis, lag time (odds ratio (OR) 1.006, 95% CI 1.002–1.010 per 1 s, *p* = 0.007) and unprovoked VTE (OR 19.61, 95% CI 1.13–59.31, *p* = 0.041) were independently associated with this adverse event in VTE patients on rivaroxaban (Table 2).

Among VTE patients on VKA, age, gender, BMI, creatinine, INR, diabetes mellitus and CAT parameters were identified as potentially associated with MBs. By multivariate analysis, peak thrombin generation (OR 0.995, 95% CI 0.989–0.999 per 1 nM, *p* = 0.045) was the only independent factor associated with this kind of bleeding on VKA (Table 2).

## 4. Discussion

We demonstrated that CAT parameters, measured prior to the next dose of rivaroxaban, could be useful in the prediction of minor bleedings in patients with VTE. We found that both lag time reflecting the time necessary to start thrombin generation as well as time to peak thrombin generation are valuable predictors of minor bleedings in patients on rivaroxaban, but not in those on VKA. Moreover, both time to start thrombin generation as well as the absence of any identifiable cause of VTE should be considered as potential predictors of elevated risk of MBs. Our findings regarding minor bleeds indicate that in the real-life setting, CAT assessment could be useful in the optimization of anticoagulation strategy in VTE patients treated on a long-term basis. Nevertheless, despite the fact that major bleedings, irrespective of the type of anticoagulation, were more frequent in patients with MB, CAT parameters were not identified as predictors of major bleedings.

MBs are commonly observed on anticoagulation and are clinically relevant, though hardly predictable, in the context of recent studies [13,14,15]. To our knowledge, studies in which the risk of bleeding was assessed using CAT have been performed solely in patients on VKA [34]. Bloemen et al. [35], in a prospective cohort study involving 129 patients on VKA, found significantly lower values of ETP and thrombin peak concentrations in patients with diverse bleeding complications. In turn, Dargaud et al. [36] showed that patients on warfarin with INR within the recommended range admitted with hemorrhage were characterized by markedly lower ETP. Our VTE patients with MBs on VKA had only lower peak thrombin generation without any significant differences after adjustment for INR in other CAT parameters. These discrepancies might be associated with both different thrombin generation assays applied as well as with different clinical and laboratory characteristics of the studied patients. In Bloemen’s study [35], significant differences were found only in the whole blood thrombin generation assay but not in plasma-based assays. Moreover, in both cited studies [35,36], INR values were higher as compared with those determined in our patients, particularly in the group without minor bleeds. Finally, the study by Dargaud et al. [36] included patients who were admitted to the emergency department with acute illnesses including major bleedings or thrombosis. In turn, of particular importance in real-life are the present findings regarding VTE patients on rivaroxaban showing association of CAT parameters with MBs during follow-up. Precisely lag time and TTP had the highest predictive value with differences also after adjustment for the residual rivaroxaban concentration. This observation is novel and might have practical implications, if corroborated in larger cohort studies.

Our unexpected finding linking unprovoked VTE to MBs in VTE patients on rivaroxaban deserves a comment. Given the recurrence rate after unprovoked VTE episodes reaching 11% at 1 year and 30% at 5 years after cessation of anticoagulation [37], in patients with low or moderate bleeding risk, indefinite anticoagulation longer than 3 months is recommended with regular bleeding risk assessment [38,39]. Prolonged treatment increases the risk of various types of bleedings and might contribute to the higher MBs incidence observed in our cohort. Moreover, patients with unprovoked VTE were older, more often male and with higher body mass index. These factors represent recognized risk factors in scales used to predict bleeding in anticoagulated VTE patients such as a VTE-BLEED score [40]. This study suggests that unprovoked VTE patients, while anticoagulated, should be screened for MBs given the risk of non-compliance in the case of persistent, or even mild, bleeding complications.

Our study has several limitations. Firstly, the sample size is relatively small, however adequately powered. The low number of major bleedings hampers analysis of their association with CAT, and we cannot exclude that CAT curves display a different pattern in larger groups of VTE patients with major bleeds compared with those without this complication, as shown previously for patients treated with warfarin [35,36]. Secondly, since our patients had rivaroxaban concentrations undetectable or lower than 100 ng/mL, it remains to be established whether CAT parameters measured at peak drug concentration might have a similar predictive value. Thirdly, we did not determine other potential modulators of blood coagulation and fibrinolysis, which might affect the CAT parameters, e.g., prothrombin, antithrombin, α2-macroglobulin or α2-antiplasmin [41]. Finally, clinical relevance of altered CAT parameters in anticoagulated VTE patients using VKA or rivaroxaban, as well as apixaban, dabigatran or edoxaban, in terms of both life-threatening bleeding as well as thromboembolic events, remains to be confirmed in larger studies.

## 5. Conclusions

In VTE patients requiring chronic anticoagulation, both time to start thrombin generation and time to peak thrombin generation derived from CAT may predict the risk of minor bleedings on rivaroxaban. Together with unprovoked etiology of VTE, time to start thrombin generation were found to be independently associated with a predisposition to minor bleedings. However, CAT parameters are not accurate enough in the prediction of minor bleedings in patients on VKA. Our findings require further validation in the larger studies.

## Figures and Tables

**Table 1:** Baseline characteristics of the studied patients.

	Controls *n* = 31	Rivaroxaban without MB *n* = 105	Rivaroxaban with MB *n* = 27	VKA without MB *n* = 120	VKA with MB *n* = 25
Age, year	42 (36–48)	46 (36–57)	47 (42–52)	47 (37–57)	50 (38–61)
Male, *n* (%)	12 (38.7)	38 (36.2)	15 (55.6)	55 (45.8)	10 (40.0)
Body mass index, kg/m^2^	25.1 (22.0–28.3)	27.3 (22.3–30.7)	26.6 (25.6–29.8)	28 (24.1–30.5) *	26.8 (23.6–30.4)
Currently smoking, *n* (%)	7 (22.6)	15 (14.3)	7 (25.9)	31 (25.8)	7 (28.0)
**VTE characteristics, *n* (%)**					
Baseline VTE diagnosis
DVT alone	15 (48.4)	40 (38.1)	9 (33.3)	43 (35.8)	12 (48.0)
Pulmonary embolism alone	6 (19.3)	25 (23.8)	9 (33.3)	28 (23.4)	6 (24.0)
Pulmonary embolism and DVT	7 (22.6)	29 (27.6)	9 (33.3)	43 (35.8)	7 (28.0)
Other	3 (9.7)	11 (10.5)	0	6 (5.0)	0
Unprovoked VTE	15 (48.4)	55 (52.3)	24 (88.9) #	67 (55.8)	11 (44.0)
Proximal DVT	16 (51.6)	62 (59.0)	16 (59.3)	71 (59.2)	19 (76)
Bilateral DVT	3 (9.7)	5 (4.8)	0	3 (2.5)	1 (4)
Time since last VTE, month	10 (6–22)	10 (6–17)	8 (6–17)	12 (8–23)	8 (7–16)
Duration of anticoagulation, month	6 (4–12)	7 (5–14)	8 (6–17)	10 (6–18)	8 (6–13)
Family history of VTE	14 (45.2)	34 (32.4)	8 (29.6)	35 (29.2)	4 (16.0)
Varices	11 (35.5)	37 (35.2)	11 (40.7)	37 (30.8)	9 (36.0)
**Comorbidities, n (%)**					
Coronary artery disease	0	3 (2.9)	0	3 (2.5)	1 (4.0)
Prior ischemic stroke	0	6 (5.7)	1 (3.7)	3 (2.5)	1 (4.0)
Hypertension	4 (12.9)	31 (29.5)	7 (25.9)	39 (32.5) *	10 (40.0)
Heart failure	1 (3.2)	1 (1.0)	0	2 (1.7)	1 (4.0)
Diabetes mellitus	2 (6.5)	4 (3.8)	0	5 (4.2)	4 (16.0) **
Chronic kidney disease	1 (3.2)	3 (2.9)	0	0	0
Use of aspirin	4 (12.9)	8 (7.6)	0	7 (5.8)	2 (8.0)
Use of statin	2 (6.5)	15 (14.3)	1 (3.7)	17 (14.2)	3 (12.0)
Proton pump inhibitor	3 (9.7)	24 (22.9)	1 (3.7) ##	25 (20.8)	4 (16.0)
**Thrombophilia, n (%)**					
Factor V Leiden	11 (35.5)	26 (24.8)	6 (22.2)	23 (19.2)	2 (8.0)
Prothrombin G20210A mutation	1 (3.2)	6 (5.7)	0	6 (5.0)	5 (20.0) **
Deficiencies in natural anticoagulants	1 (3.2)	9 (9.6)	3 (11.1)	16 (13.3)	7 (28.0)
Antiphospholipid syndrome	2 (6.5)	7 (6.7)	5 (18.5)	10 (8.3)	2 (8.0)
**Laboratory Investigations**					
White blood cells, 10^3^/μL	5.90 (4.92–7.54)	5.89 (4.85–7.1)	6.07 (5.69–6.94)	6.36 (5.27–7.66)	6.08 (5.46–7.19)
Hemoglobin, g/dL	13.6 (12.9–14.5)	13.9 (13.1–14.9)	13.9 (13.1–15.4)	14.4 (13.4–15.5) *	13.4 (13.1–14.5)
Platelets, 10^3^/μL	232 (195–265)	245 (206–284)	216 (186–265)	244 (201–285)	243 (212–293)
Glucose, mmol/L	5.1 (4.8–5.3)	5.1 (4.9–5.8)	5.2 (5.0–5.4)	5.2 (4.9–5.6)	5.4 (5.0–5.7)
Creatinine, μmol/L	70 (64–85)	70 (61–79)	74 (67–89)	72 (65–83)	70 (64–82)
eGFR, ml/min/1.73 m^2^	92 (83–114)	101 (89–111)	94 (86–106)	98 (88–107)	99 (90–1017)
hsCRP, ng/mL	1.12 (0.71–1.87)	1.1 (0.6–3.43)	1.98 (0.9–5.6)	1.76 (0.85–3.11)	1.52 (0.74–3.58)
D-Dimer, ng/mL	275 (183–447)	265 (171–375)	209 (171–348)	203 (171–341)	177 (171–226)
Rivaroxaban concentration, μg/L	-	25 (13–44)	35 (6–66)	-	-
APTT, s	25.8 (24.7–29.1)	25.8 (24.1–28.4)	27.1 (24.9–34.1)	28.7 (25.1–34.1) *	31.0 (25.4–35.7)
INR	0.99 (0.97–1.04)	1.03 (0.99–1.14)	1.04 (0.98–1.10)	1.47 (1.02–2.20)	2.19 (1.46–2.45) **
Fibrinogen, g/L	3.03 (2.61–4.0)	3.06 (2.64–3.64)	3.3 (2.8–4.05)	3.13 (2.71–363)	3.24 (2.91–3.48)

Abbreviations: data are shown as numbers (percentages) or median (interquartile range), DVT: deep vein thrombosis, eGFR: glomerular filtration rate, MB: minor bleeding, VKA: vitamin K antagonist, VTE: venous thromboembolism, APTT: activated partial thromboplastin time, INR: international normalized ratio. There were no significant differences between the following groups: (1) controls and patients on rivaroxaban without MB, (2) controls and patients on VKA without MB, (3) patients on rivaroxaban with MB and without MB, and (4) patients on VKA with MVB and without MB. The only significant intergroup differences were marked as follows: * *p* < 0.05 vs. controls, ** *p* < 0.05 vs. VKA without MB, # *p* < 0.001 vs rivaroxaban without MB, ## *p* < 0.05 vs rivaroxaban without MB.

**Table 2:** The independent predictors of minor bleedings on rivaroxaban or on VKA.

	Univariate Model			Multivariate Model		
Independent Variable	*p*-Value	OR	95% CI for OR	*p*-Value	OR	95% CI for OR
**MB on Rivaroxaban**						
Age, per 1 year	0.586	1.010	0.975–1.045	0.996	1.000	0.929–1.076
Male gender, yes/no	0.071	0.454	0.193–1.069	0.828	0.818	0.134–5.005
Body mass index, per 1 kg/m^2^	0.935	0.997	0.927–1.073	0.787	0.977	0.827–1.155
Creatinine, per 1 μmol/L	0.069	1.029	0.998–1.061	0.503	0.980	0.926–1.039
INR, per 0.01	0.427	0.982	0.940–1.026	0.542	0.969	0.877–1.071
Rivaroxaban concentration, per 1 μg/L	0.349	1.011	0.989–1.033	0.947	1.001	0.970–1.034
Unprovoked VTE, yes/no	0.002	7.246	2.062–25.641	0.041	19.607	1.131–59.311
Lag time, per 1 s	<0.001	1.004	1.002–1.006	0.007	1.006	1.002–1.010
**MB on VKA**						
Age, per 1 year	0.811	1.004	0.969–1.042	0.851	0.996	0.956–1.038
Male gender, yes/no	0.594	1.269	0.528–3.051	0.928	0.948	0.297–3.024
Body mass index, per 1 kg/m^2^	0.971	0.998	0.917–1.088	0.972	0.998	0.905–1.101
Creatinine, per 1 μmol/L	0.563	0.991	0.960–1.022	0.365	0.980	0.938–1.024
INR, per 0.01	0.027	1.007	1.001–1.013	0.054	1.006	1.000–1.013
Diabetes mellitus, yes/no	0.038	4.386	1.086–17.544	0.059	4.098	0.948–17.857
Max IIa, per 1 nM	0.023	0.994	0.989–0.999	0.045	0.995	0.989–0.999

Abbreviations: OR: odds ratio, CI: confidence interval, MB: minor bleeding, INR: international normalized ratio, VTE: venous thromboembolism, VKA: vitamin K antagonist, lag time: time to start thrombin generation, max IIa: peak thrombin generation. For model with rivaroxaban Negelkerke R^2^ was 0.49 and for VKA 0.16 (*p* < 0.001 for both).

## Supplementary Materials

The following are available online at https://www.mdpi.com/2077-0383/9/7/2018/s1, Table S1. Minor and major bleedings in the studied groups during follow-up, Table S2: The calibrated automated thrombography parameters, Table S3: Baseline characteristics of patients on rivaroxaban with provoked and unprovoked VTE.
