# Supplementary material for: Delayed Thrombin Generation Is Associated with Minor Bleedings in Venous Thromboembolism Patients on Rivaroxaban: Usefulness of Calibrated Automated Thrombography
Source: J Clin Med. 2020 Jun 27;9(7):2018. doi: 10.3390/jcm9072018 (PMC7409038; doi:10.3390/jcm9072018)
Supplement: Supplementary file 1 [file jcm-09-02018-s001.zip › Table S3.docx]

**Table S3.** Baseline characteristics of patients on rivaroxaban with provoked and unprovoked VTE.

|  | **Unprovoked VTE**  **N=79** | **Provoked VTE**  **N=53** | **P-value** |
| --- | --- | --- | --- |
|  |  |  |  |
| Age, y | 50 (43-61) | 40 (32-48) | <0.001 |
| Male, n (%) | 45 (57.0) | 8 (15.1) | <0.001 |
| Body mass index, kg/m^2^ | 27.8 (24.6-30.9) | 25.1 (21.3-29.4) | 0.007 |
| Current smoking, n (%) | 16 (20.3) | 6 (11.3) | 0.24 |
| Baseline VTE diagnosis |  |  | 0.66 |
| DVT alone, n (%) | 26 (32.9) | 23 (43.4) |  |
| Pulmonary embolism alone, n (%) | 21 (26.6) | 13 (24.5) |  |
| Pulmonary embolism and DVT, n (%) | 25 (31.6) | 13 (24.5) |  |
| Other, n (%) | 7 (8.9) | 4 (7.6) |  |
| Proximal DVT, n (%) | 56 (70.9) | 36 (67.9) | 0.72 |
| Bilateral DVT, n (%) | 2 (2.5) | 0 | 0.24 |
| Time since last VTE, month | 10 (6-17) | 10 (6-15) | 0.64 |
| Duration of anticoagulation, month | 8 (6-16) | 7 (5-12) | 0.38 |
| Family history of VTE, n (%) | 24 (30.4) | 18 (34.0) | 0.71 |
| Varices, n (%) | 33 (41.8) | 15 (28.3) | 0.14 |
| Coronary artery disease, n (%) | 2 (2.5) | 1 (1.9) | 0.99 |
| Prior ischemic stroke, n (%) | 5 (6.3) | 2 (3.8) | 0.70 |
| Hypertension, n (%) | 28 (35.4) | 10 (18.9) | 0.05 |
| Heart failure, n (%) | 1 (1.3) | 0 | 0.99 |
| Diabetes mellitus, n (%) | 3 (3.8) | 1 (1.9) | 0.31 |
| Chronic kidney disease, n (%) | 0 | 3 (5.7) | 0.06 |
| Use of aspirin, n (%) | 4 (5.1) | 4 (7.5) | 0.71 |
| Use of statin, n (%) | 10 (12.7) | 6 (11.3) | 0.98 |
| Use of ACEI or ARB | 19 (24.1) | 5 (9.4) | 0.039 |
| Proton pump inhibitor | 15 (19.0) | 10 (18.9) | 0.99 |
| White blood cells, 10^3^/µL | 5.93 (5.40-7.06) | 5.99 (4.83-7.10) | 0.56 |
| Hemoglobin, g/dL | 14.2 (13.4-15.6) | 13.5 (12.7-14.0) | <0.001 |
| Platelets, 10^3^/µL | 216 (188-276) | 261 (222-287) | 0.004 |
| Glucose, mmol/L | 5.3 (5.0-5.8) | 5.0 (4.8-5.3) | 0.002 |
| Creatinine, µmol/L | 76 (65-86) | 64 (58-74) | <0.001 |
| eGFR, ml/min/1.73 m^2^ | 95 (86-106) | 107 (95-116) | 0.003 |
| hsCRP, ng/mL | 1.35 (0.62-3.80) | 1.13 (0.62-4.15) | 0.99 |
| D-Dimer, ng/mL | 278 (181-377) | 228 (171-375) | 0.50 |
| Fibrinogen, g/L | 3.14 (2.68-3.85) | 3.03 (2.64-3.58) | 0.22 |
| INR | 1.04 (0.98-1.11) | 1.02 (0.99-1.16) | 0.95 |
| Lag time, s | 589 (399-760) | 494 (323-808) | 0.19 |
| TTP, s | 874 (665-1159) | 760 (551-1121) | 0.16 |
| Max IIa, nM | 191 (137-240) | 220 (156-266) | 0.048 |
| Max rate, nM/s | 1.7 (1.2-2.1) | 1.8 (1.4-2.3) | 0.30 |
| ETP, nM×s | 138217 (116851-157672) | 151033 (127204-171368) | 0.018 |

Abbreviations: data are shown as numbers (percentages) or median (interquartile range), ACEI: angiotensin converting enzyme, ARB: angiotensin receptor blocker, ETP: endogenous thrombin potential, DVT: deep vein thrombosis, eGFR: glomerular filtration rate, hsCRP: high sensitivity C-reactive protein, INR: international normalized ratio, lag time: time to start thrombin generation, max IIa: peak thrombin generation, max rate: the highest rate of thrombin generation, TTP: time to peak thrombin generation, VTE: venous thromboembolism.
