# Supplementary material for: Delayed Thrombin Generation Is Associated with Minor Bleedings in Venous Thromboembolism Patients on Rivaroxaban: Usefulness of Calibrated Automated Thrombography
Source: J Clin Med. 2020 Jun 27;9(7):2018. doi: 10.3390/jcm9072018 (PMC7409038; doi:10.3390/jcm9072018)
Supplement: Supplementary file 1 [file jcm-09-02018-s001.zip › Table S1.docx]

**Table S1.** Minor and major bleedings in the studied groups during follow-up.

|  | **Type of bleeding** | **Controls**  **N=31** | **Rivaroxaban**  **N=132** | **VKA**  **N=145** |
| --- | --- | --- | --- | --- |
| Minor bleeding | All | 2 (6.4) | 27 (20.5) | 25 (17.2) |
|  | Skin easy bruising | 0 | 8 (6.1) | 8 (5.5) |
|  | Gingival | 1 (3.2) | 2 (1.5) | 6 (4.1) |
|  | Epistaxis | 0 | 5 (3.8) | 3 (2.1) |
|  | Hematuria | 0 | 4 (3.0) | 2 (1.4) |
|  | Conjunctival bleeds | 1 (3.2) | 1 (0.8) | 2 (1.4) |
|  | Vaginal | 0 | 7 (5.3) | 4 (2.8) |
| Major bleeding | All | 0 | 7 (5.3) | 6 (4.1) |
|  | Gastrointestinal | 0 | 5 (3.8) | 2 (1.4) |
|  | Vaginal | 0 | 0 | 2 (1.4) |
|  | Menorrhagia | 0 | 1 (0.8) | 0 |
|  | Others | 0 | 1 (0.8) | 2 (1.4) |

Abbreviations: data are shown as numbers (percentages), VKA: vitamin K antagonist.
