# Supplementary material for: Delayed Thrombin Generation Is Associated with Minor Bleedings in Venous Thromboembolism Patients on Rivaroxaban: Usefulness of Calibrated Automated Thrombography
Source: J Clin Med. 2020 Jun 27;9(7):2018. doi: 10.3390/jcm9072018 (PMC7409038; doi:10.3390/jcm9072018)
Supplement: Supplementary file 1 [file jcm-09-02018-s001.zip › Table S2.docx]

**Table S2.** The calibrated automated thrombography parameters.

|  | Controls**  N=31 | Rivaroxaban without MB*  N=105 | Rivaroxaban with MB*  N=27 | VKA without MB**  N=120 | VKA with MB  N=25 |
| --- | --- | --- | --- | --- | --- |
|  |  |  |  |  |  |
| Lag time, s | 399 (266-504) | 475 (323-646) | 817 (722-988) | 627 (360-1002) | 760 (450-1406) |
| TTP, s | 627 (523-732)# | 741 (570-969)# | 1178 (1045-1311) | 922 (589-1259) | 1235 (703-1634) |
| Max IIa, nM | 233 (201-305) | 220 (169-261) | 134 (78-191) | 172 (78-254)## | 104 (59-194)## |
| Max rate, nM/s | 2.2 (1.7-2.7) | 1.9 (1.5-2.2) | 1.2 (0.9-1.6) | 1.6 (1-2.2) | 1.1 (0.8-1.9) |
| ETP, nMxs | 156313 (142350-169853) | 149036 (127389-168382) | 125101 (61859-141591) | 116184 (62957-158276) | 88447 (50950-132937) |

Abbreviations: data are shown as median (interquartile range), ETP: endogenous thrombin potential, max IIa: peak thrombin generation, max rate: the highest rate of thrombin generation, MB: minor bleeding, lag time: time to start thrombin generation, TTP: time to peak thrombin generation, VKA: vitamin K antagonist, *P<0.001 for difference between each CAT parameter in compared groups adjusted for rivaroxaban concentration, **P<0.001 for difference between each CAT parameter in compared groups adjusted for INR, #P<0.05 for difference between indicated parameter in compared groups adjusted for rivaroxaban concentration, ##P<0.05 for difference between indicated parameter in compared groups adjusted for INR.
